# Ion Channel Trafficking: Control of Ion Channel Density as a Target for Arrhythmias?

**DOI:** 10.3389/fphys.2017.00808

**Published:** 2017-10-17

**Authors:** Elise Balse, Hannah E. Boycott

**Affiliations:** ^1^Unité de Recherche sur les Maladies Cardiovasculaires, le Métabolisme et la Nutrition, Faculté de Médecine Pitié-Salpêtrière, Sorbonne Universités, UPMC Univ. Paris VI, Inserm, UMRS 1166, Université Pierre et Marie Curie, Paris, France; ^2^Department of Cardiovascular Medicine, John Radcliffe Hospital, University of Oxford, Oxford, United Kingdom

**Keywords:** potassium channels, sodium channel, arrhythmias, cardiac, trafficking, accessory proteins

## Abstract

The shape of the cardiac action potential (AP) is determined by the contributions of numerous ion channels. Any dysfunction in the proper function or expression of these ion channels can result in a change in effective refractory period (ERP) and lead to arrhythmia. The processes underlying the correct targeting of ion channels to the plasma membrane are complex, and have not been fully characterized in cardiac myocytes. Emerging evidence highlights ion channel trafficking as a potential causative factor in certain acquired and inherited arrhythmias, and therapies which target trafficking as opposed to pore block are starting to receive attention. In this review we present the current evidence for the mechanisms which underlie precise control of cardiac ion channel trafficking and targeting.

## Functional expression of ion channels in the sarcolemma and cardiac excitability

The function of the heart is governed by the electrical and mechanical activity of myocytes. The functional expression of several different types of ion channels in the myocyte sarcolemma determines the shape and duration of the action potential (AP), and therefore controls the effective refractory period (ERP) of the myocardium. The ERP is a protective mechanism that keeps the heart rate in check and thus prevents arrhythmias. Any prolongation or shortening of the ERP is therefore potentially arrhythmogenic. Most genetic arrhythmias are caused by mutations which alter the biophysical properties of ion channels. However, the proper functional expression of ion channels can be disrupted at several points including at the transcriptional, translational, and post-translational levels. In the last two decades, studies have emerged in which mutations carried by ion channels have been shown to be linked to trafficking defects, resulting in retention and/or degradation of the channel early in the trafficking process.

**Figure 1:**
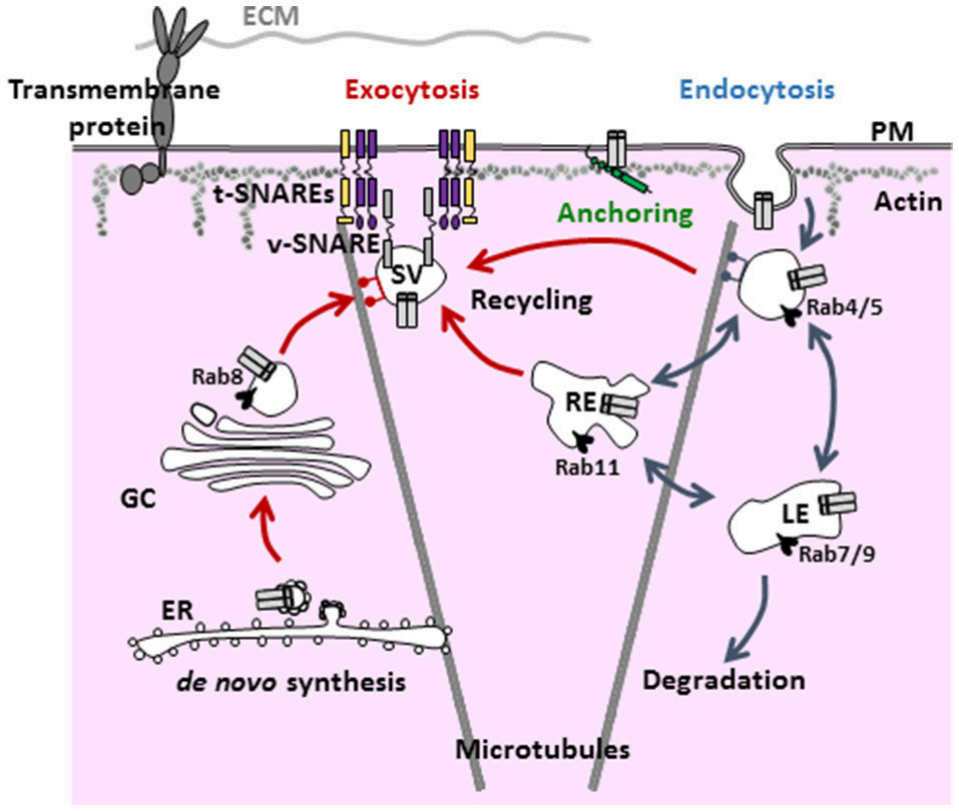
General scheme of the various steps and regulators involved in the trafficking of ion channels. Ion channels are targeted to the plasma membrane via the anterograde and recycling pathways (red arrows). Once targeted to a specialized domain of the membrane, ion channels are stabilized by anchoring partners/associate subunits to be functional (green). Then, signals for internalization (blue arrows) lead to either degradation or recycling. PM, plasma membrane; EE, early endosome; LE, late endosome; ECM, extracellular matrix; RE, recycling endosome; GC, Golgi complex; ER, endoplasmic reticulum; SV, secretory vesicle.

The density of active ion channels in specific membrane domains is a dynamic process resulting from the concomitant and antagonistic action of anterograde (exocytosis, recycling) and retrograde (internalization) pathways. Targeting and stabilization of these channels by anchoring partners in specialized domains of the sarcolemma also dynamically regulate the electrical activity of the cell (Balse et al., 2012; Figure 1).

The majority of mutations related to trafficking defects involve endoplasmic reticulum (ER) exit defects, leading to targeting of misfolded channels to degradation by the ERAD system (endoplasmic reticulum-associated degradation, lysosomal, or proteasomal). Such mutations have been identified for *HERG* (Furutani et al., 1999), *KCNQ1* (Gouas et al., 2004), and *SCN5A* (Valdivia et al., 2004) and associated with long QT and Brugada syndromes. Sorting signals carried by ion channels also are necessary for ER and trans-Golgi network exit (TGN) (Kupershmidt et al., 2002). A mutation involving an endocytotic defect of *TRPM4* has also been identified in human progressive familial heart block type I (Kruse et al., 2009). Finally, another crucial factor is the association (early or late) of ion channels with ancillary subunits, chaperones, and anchoring partners that are involved in channel function and localization.

## Beyond channelopathies: trafficking processes as regulators of cardiac action potential

Trafficking and targeting of integral membrane proteins, including ion channels and receptors, have been explored in other cell types such as epithelial cells and neurons. Although these studies have yielded great insights, these mechanisms have not been studied extensively in cardiomyocytes. Such research may provide insight into the regulation of cardiac excitability *via* ion channel trafficking. Although trafficking defects have been reported for certain ion channel mutations, and are associated with retention in intracellular organelles, the characterization of intracellular trafficking in control and disease conditions are largely lacking in native cardiomyocytes. The fact that these cells are highly structurally and functionally specialized suggests that trafficking and targeting of cardiac ion channels may involve unique and specific pathways. Furthermore, the propagation of electrical activation within the myocardium is a complex process controlled by the spatial/differential distribution of ion channels within single cardiomyocytes that eventually establishes the anisotropic ratio. The underlying mechanisms regulating ion channel targeting to sarcolemmal subdomains, and tethering into macromolecular complexes remains a largely unanswered, yet important question.

In acquired arrhythmias such as heart failure and atrial fibrillation, ion channel dysfunction and electrical remodeling are often associated with tissue remodeling including hypertrophy/dilatation, replacement and interstitial fibrosis, and gap junction disorganization (Rucker-Martin et al., 2006). In this context, abnormal trafficking and targeting of cardiac ion channels emerge as important pathogenic factors of the electrical remodeling (Schotten et al., 2011). Importantly, increased knowledge of the pathways underlying cardiac ion channel trafficking may yield novel drug targets which lack the problems associated with conventional pore block therapies. Such therapies are frequently highly non-selective and have numerous unwanted side effects. The purpose of this mini-review is to provide an overview of the mechanisms of trafficking of ion channels in native cardiomyocytes that could potentially result in the discovery of new targets for antiarrhythmic therapies.

## Hypokalemia and hERG channel regulation

The I_Kr_ current is encoded by the human ether-a-go-go–related gene (*HERG*, also known as *KCNH2*) in the heart (Sanguinetti et al., 1995; Trudeau et al., 1995). *HERG* mutations resulting in reduced I_Kr_ cause type 2 long QT syndrome (LQT2), which predisposes individuals to life-threatening arrhythmias. *HERG* mutations often disrupt the forward trafficking of hERG (thereby reducing sarcolemmal expression of the channel), and subsequently result in decreased *I*_Kr_ (Anderson et al., 2006). The hERG channel is also a notorious target for several classes of drugs that engender acquired long QT syndrome (LQTS) (Sanguinetti and Tristani-Firouzi, 2006). For instance, LQTS and Torsades de pointes are exacerbated by hypokalemia, with a moderate increase in serum [K^+^] capable of correcting LQTS in some patients (Compton et al., 1996). Hypokalemia is therefore considered a risk factor for LQTS and sudden cardiac death.

Guo and colleagues elegantly revealed how the plasma membrane density of hERG channels is regulated under physiological and pathophysiological (hypokalemia) conditions. They showed that lowering extracellular potassium drastically accelerated hERG internalization and degradation both in overexpression systems and native *I*_Kr_ in a hypokalemia rabbit model (Guo et al., 2009). Rabbits fed with low-K^+^ diet showed prolonged QTc correlated to significantly prolonged APD_90_. *In vitro*, exposure to 0 mM K^+^ medium completely and reversibly eliminated *I*_Kr_ without significant effects on other potassium currents. The decrease in *I*_Kr_ accompanied reduced expression of hERG, combined with reduced surface expression of the channel. Finally, they showed that 0 mM K^+^ medium induced the internalization of hERG by increasing ubiquitylation and resulted in lysosomal degradation (Guo et al., 2009). These findings provide a potential mechanism for hypokalemia-induced exacerbation of LQTS.

## Lipidic content and K_V_1.5 channel regulation

Cardiac excitability can be regulated by cellular lipid content. Free cholesterol is a major lipid class shown to regulate membrane fluidity, curvature, and stiffness (Lundbaek et al., 1996). The function of several cardiac ion channels are regulated directly by cholesterol, which modulates channel properties (Oliver et al., 2004; Epshtein et al., 2009).

The *I*_Kur_ current, carried by K_V_1.5 channels, is an important component of atrial repolarization (Fedida et al., 1993; Wang et al., 1993) and has been implicated in the pathology of atrial fibrillation (Van Wagoner et al., 1997; Brundel et al., 2001). The groups of D. Fedida and J. Martens contributed substantially to the study of the trafficking of K_V_1.5 channel in expression systems (McEwen et al., 2007; Steele et al., 2007). Trafficking of vesicles is regulated by several Rab GTP-ases, involved at every stage of the process; regulating sorting, tethering and docking of trafficking vesicles. The early endosome (EE), associated with Rab4, mediates fast recycling while the recycling endosome, associated with Rab11, coordinates the slow recycling of proteins back to the cell membrane.

We showed that membrane cholesterol depletion *via* methyl-β-cyclodextrin (MβCD) increased the number of functional K_V_1.5 channels in the sarcolemma of atrial myocytes, also reducing their mobility as shown by Fluorescence recovery After Photobleaching (Balse et al., 2009). Cholesterol depletion triggers exocytosis of ion channels from sub-membrane compartments. Indeed, the overexpression of a dominant negative (DN) form of Rab11 (associated with the recycling endosome), prevented the current increase upon MβCD treatment whereas overexpression of a DN form of Rab4, associated with the early endosome, did not. The recycling endosome, considered a slow route for ion channel recycling (Steele et al., 2007), is particularly sensitive to cholesterol depletion as rapid dissociation (<10 min) of the channel from the vesicle was observed (Balse et al., 2009). These results showed that channel turnover can be modified by changes in the lipid environment and that sub-membrane storage compartments can be recruited to modify the electrical properties of cardiomyocytes.

## Mechanical challenge and K_V_1.5 channel regulation

Cardiomyocytes are exposed to mechanical forces when the heart contracts. These forces consist of stretch, shear and strain constraints. Shear forces in the myocardium are primarily generated by the movement of sheets of cardiomyocytes sliding relative to each other when the muscle contracts, as well as by blood flow during the cardiac cycle (LeGrice et al., 1995; Costa et al., 1999). Increased shear stress stimulates intracellular calcium transients (Morad et al., 2005), increases the beating rate of neonatal ventricular myocytes (Lorenzen-Schmidt et al., 2006), and triggers propagating APs in monolayers of ventricular myocytes (Kong et al., 2005).

We showed that in rat atrial myocytes shear stress activates a large outward current, mirrored by a decrease in AP duration (Boycott et al., 2013). The main ion channel mediating the increase in current K_V_1.5, which was recruited from subcellular compartments to the sarcolemma, a phenomenon which was directly observed by TIRF microscopy. The donor compartment was again identified as the recycling endosome. K_V_1.5 channel exocytosis requires integrin signaling through focal adhesion kinase (FAK) and relies on an intact microtubule system. We also found that the response was dysregulated in a model of chronic hemodynamic overload. Hypertrophied atrial myocytes had reduced K_v_1.5 expression, despite an increase in basal *I*_*Kur*_. The response of these cardiomyocytes to shear stress was reduced, and the kinetics altered. Our results suggested chronically increased mechanical stress over activates the integrin signaling pathway, resulting in an increased *I*_*Kur*_, AP shortening and a reduction in the capability of cells to respond to shear stress (Boycott et al., 2013). Thus, pools of K_V_1.5 from the slow recycling route may comprise an inducible reservoir mediating faster atrial repolarization. The shortening of the AP observed following heart failure in the atria could be partly explained by a shift in the trafficking balance toward increased exocytosis.

## Modulation of K_V_1.5 internalization by antiarrhythmics

Whereas conventional antiarrhythmic drugs generally target ion permeability by binding to the pore of the channel, increasing evidence suggests some compounds can indirectly disrupt protein trafficking. In this context, sustained efforts have been conducted to develop antiarrhythmic agents that affect channel trafficking, notably hERG channels blockers that stabilize misfolded channels and rescue hERG trafficking mutants (Wible et al., 2005).

The group of J. Martens has been at the forefront of investigations into the potential antiarrhythmic properties of drugs which acutely modulate the surface density of functional channels (Schumacher et al., 2009). The class I antiarrhythmic drug quinidine is known to inhibit *I*_Kur_ current through open-channel block of K_V_1.5 channel. Using an immunocytochemical approach to quantify surface and internalized channels, Schumacher and colleagues showed that quinidine dose-dependently induced the internalization of K_V_1.5 channel over short-time frames (10 min) in the HL-1 cell line and in dissociated neonatal mouse myocytes. The effect of quinidine was stereospecific as quinine has no effect on K_V_1.5 channel surface density (despite exerting the pore-block effect of its stereoisomer) and subunit-dependent, as the K_V_1.5-related channels K_V_2.1 or K_V_4.2 did not show internalization at the same time points and doses. In addition, quinidine-induced K_V_1.5 channel internalization followed the same endocytotic pathway as constitutive endocytosis (as previously identified Choi et al., 2005), being microtubule-dependent and dynamin-mediated. Finally, whereas acute treatment allows channel recycling to the surface, chronic treatment with a clinical-compatible concentration led to channel degradation through the proteasome. This pharmacological control of K_v_1.5 surface expression and trafficking represents a novel mechanism by which drug stimulated endocytosis of an ion channel may be utilized as an anti-arrhythmic tool.

## Organization of ion channel macromolecular complexes

It is noteworthy that emerging evidence challenges the assumption that ion channels function as homogeneous complexes. Rather, it seems likely that ion channels form macro-complexes with other ion channels, and that these complexes contribute to the stabilization of channels at the sarcolemma, and facilitate proper electrical conduction. The MAGUK protein SAP97 contributes to the formation of macromolecular complexes involving different ion channel families: K_V_1.5 (Godreau et al., 2002; Abi-Char et al., 2008), K_V_4.x (El-Haou et al., 2009; Gillet et al., 2015), Kir2.x (Leonoudakis et al., 2001; Milstein et al., 2012; Matamoros et al., 2016), and Na_V_1.5 (Petitprez et al., 2011; Milstein et al., 2012), SAP97 also couples ion channels to signaling pathways, allowing the regulation of K_V_4.x channels by CaMKII (El-Haou et al., 2009). An important recent breakthrough showed that SAP97 regulates the formation of Na_V_1.5/Kir2.1 complexes, the two critical channels underlying *I*_K1_ and *I*_Na_, respectively. These currents are responsible for maintenance of the resting membrane potential and rapid depolarization during the upstroke of the AP (Milstein et al., 2012). This multi-channel organization enables reciprocal modulation, contributing to maintenance of normal cardiac excitability. Interestingly, co-expression with Na_v_1.5 seems to reduce internalization of Kir2.1, suggesting that participation in a macromolecular complex reduces anterograde trafficking of Kir2.1 (Milstein et al., 2012). Another example of multi-channel organization for Na_V_1.5/Kir2.x, but mediated by syntrophin, has been recently reported (Matamoros et al., 2016). In addition to Na_v_1.5, other cardiac ion channels have been shown to interact. For example, the KCNQ1/KCNE1 genes that encode *I*_Ks_ (Barhanin et al., 1996; Sanguinetti et al., 1996) interact with hERG, and the channels can reciprocally regulate (Ehrlich et al., 2004; Ren et al., 2010; Organ-Darling et al., 2013), with KCNQ1 also functioning as a chaperone for hERG trafficking (Biliczki et al., 2009). In the context of hypokalemia, KCNQ1/hERG co-expression slowed the internalization of mature, i.e. cell-surface expressed, hERG channels whereas KCNQ1 alone is not sensitive to hypokalemia (Guo et al., 2011). As the association between hERG and KCNQ1 only occurs at the plasma membrane, KCNQ1 likely contributes to hERG membrane stability.

## Modulation of ion channel anterograde trafficking by partner proteins

A fascinating question relates to the nature of the molecular mechanisms which regulate ion channel targeting into distinct subdomains of the sarcolemma and their tethering in large molecular complexes. Cardiac myocytes are structurally and functionally highly polarized cells. While the transmission of the AP between myocytes occurs at the intercalated disc (ID), the AP is conducted along the myocyte at the lateral membrane (LM).

The direct targeting of hemichannels to the ID *via* the microtubule plus-end-tracking protein EB1 (Shaw et al., 2007) and the targeting of Ca_V_1.2 to T-tubules by BIN1, a protein involved in membrane invagination and endocytotic processes (Hong et al., 2012) have been important major discoveries.

Recently, the spatial distribution of Na_V_1.5 has received interest. Whereas Na_V_1.5 is highly concentrated at the ID (3 to 8 fold larger current), Na_V_1.5 channels at the LM show a lower density (Verkerk et al., 2007; Lin et al., 2011). This differential distribution of Na_V_1.5 channels favors the anisotropic conduction of the myocardial depolarization wave (Spach, 1999). Several partners of Na_v_1.5 have been identified such as gap junctional (i.e., connexin-43), desmosomal (plakophilin-2), actin cytoskeleton-binding (ankyrin-G), and the MAGUK protein SAP97 at the ID and syntrophin at the LM (for review see Shy et al., 2014). All these partners exert a positive regulatory effect on *I*_Na_ since their silencing *in vitro*, Knock Out *in vivo* or reduced expression in the Duchenne muscular dystrophy mouse model leads to reduced sodium current and localization. However, whether these proteins are involved anterograde trafficking of Na_V_1.5 or associate with the channel once inserted in the plasma membrane to stabilize it is not clear. Recently, we have characterized a new partner of the Na_V_1.5 channel in the myocardium, the MAGUK protein CASK. CASK is located at the LM, in association with the syntrophin/dystrophin complex. In contrast with other Na_V_1.5 partners, CASK negatively regulates *I*_Na_ by impeding Na_V_1.5 anterograde trafficking to the LM (Eichel et al., 2016). In dilated human atria, associated with AF or valve regurgitation, expression of CASK is reduced without affecting its localization. The consequence of this would likely be increased *I*_Na_ at the LM and could be considered detrimental for cardiac anisotropy. Keeping with the recent discovery that SAP97 is necessary for the interaction between Na_V_1.5 and Kir2.1 (Milstein et al., 2012), MAGUK proteins could be major partners for the organization of multi-channel complexes.

## Conclusion

Although only proven *in vitro* at present, early ER/Golgi trafficking rescue has been shown to be feasible for trafficking-defective mutant channels by temperature decrease, Hsp70 and 91 cytosolic chaperones, and pharmacological chaperones. Pharmacological chaperones include channel blockers, such as anti-histamines (Astemizole), anti-serotoninergic (Cisapride) and antiarrhythmics (e.g., E-4031 for hERG), that are difficult to manipulate as they can also block the channel and worsen the disease. However, certain pore-blocking drugs, such as fexofenadine, have been shown to rescue hERG trafficking of some mutant channels at concentrations far below the IC50 for pore-block (Rajamani et al., 2002). Furthermore, in an iPSC model of LQTS2 in which hERG was not expressed at the sarcolemma, sarcolemmal hERG expression was achieved using the proteasomal inhibitor ALLN, which allowed the re-trafficking and functional expression of the channel (Mehta et al., 2014). These studies highlight the relevance of research into pharmacological rescue of trafficking deficient mutant channels. Low molecular weight compounds such as DMSD, TMD and glycerol act as nonspecific chemical chaperones mostly likely by stabilizing proteins during folding and maturation (Kaufman and Ficker, 2003). In acquired cardiopathies, three pathways are worthy of special attention: the recycling endosome/Rab11 pathway, the endocytosis/dynamin/microtubule pathway and, the association of ion channels with specific partners for anterograde trafficking and correct targeting to specialized membrane subdomains of the myocyte. Future studies are necessary to explore the specific roles of ion channel partners on ion channel trafficking/targeting vs. membrane scaffolding to understand the relative contribution of these proteins in dictating cardiac excitability and function under normal and pathological conditions. The highly specialized architecture of the cardiomyocyte dictates that more studies in native cells are necessary to improve our understanding of the mechanisms involved, and how they impact upon the pathology of arrhythmia.

## Author contributions

All authors listed have made a substantial, direct and intellectual contribution to the work, and approved it for publication.

### Conflict of interest statement

The authors declare that the research was conducted in the absence of any commercial or financial relationships that could be construed as a potential conflict of interest.
